# The study of chromosomal abnormalities and heteromorphism in couples with 2 or 3 recurrent abortions in Shahid Beheshti Hospital of Hamedan 

**Published:** 2013-03

**Authors:** Atefeh Asgari, Safieh Ghahremani, Solmaz Saeedi, Ebrahim Kamrani

**Affiliations:** 1 *Islamic Azad University, Science and Research Branch, Tehran, Iran.*; 2 *Cytogenetic Laboratories, Shahid Beheshti Hospital, Hamedan University of Medical Sciences, Hamedan, Iran.*

**Keywords:** *Translocation*, *Heteromorphism*, *Recurrentabortion*, *Chromosomal abnormalities*

## Abstract

**Background: **Different studies show that chromosomal balance translocation in the parents can cause recurrent spontaneous abortions. Incidence of chromosomal translocation abnormalities in couples with repeated abortions is from 0% to 31%.

**Objective:** The purpose of this research was studying the presence or absence of chromosomal abnormalities and heteromorphism in couples with recurrent abortions and also the role of this anomaly in the abortions.

**Materials and Methods:** This study is a cross sectional descriptive study which have investigated 75 couples who had three abortions or more, and 65 couples who had two abortions that referred by gynecologist to the lab of Beheshti Hospital in Hamedan for cytogenetical investigation. Also 40 healthy individuals without history of abortion investigated as control group.GTG bonding technique (staining banding with gymsa and trypsin) is used in this study.

**Results:**Frequency of chromosomal abnormalities and heteromorphism among couples with three or more abortions were reported respectively 5.3% and 9.3%. This frequency in couples with two abortions was respectively 3.07%and 6.15%. The frequency of chromosomal heteromorphism in control group was 7.5% and no chromosomal abnormalities were observed in them.

**Conclusion:**This study shows that chromosomal abnormality can be one reason of recurrent spontaneous abortions and more abortion increases the probability of this anomaly. Also, existence of chromosomal heteromorphism in the general population without clinical abortion symptoms shows that chromosomal heteromorphism cannot be the reason of these spontaneous abortions.

## Introduction

Generally, loss of pregnancy without outside intervention before 20^th^ week of pregnancy is called spontaneous abortion. Pregnancy miscarriage is a great problem which is really important for the couples who decide to have a baby. Recurrent spontaneous abortion in human is a general phenomenon and cytogenetical anomaly is one important reason for it (1). 

Development of banding techniques in 1970 simplified identifying chromosomal abnormalities. These techniques provide the ability to identify individual chromosomes certainly and show that each loss or gain, even a small piece of a chromosome, can have detrimental effects on human development (2). At the first time, Carr in 1960 reported that about 50% of first trimester abortions are due to structural or numerical chromosome abnormalities. Also, Kajii in his study showed that 82 samples of 152 spontaneous abortions were with chromosomal anomalies (54%), and unbalanced translocation has been reported in only 1% of these abnormalities and parental balanced translocation shows a high rate in spontaneous abortions (3, 4).

Incidence of spontaneous abortions in clinically diagnosed pregnancies was 15% to 20% while the loss of embryo after implantation in pregnancy is four times more and lost undiagnosed pregnancies are estimated two to three times more than this amount. 

More extensive studies show that excreted embryos in second trimester have 50-60% abnormalities (5). As Alberman showed in his paper, the incidence of chromosomal anomalies is reduced from 60% to 5% in 6^th^ month of pregnancy (6). Kim Show that after fertilization the incidence of chromosomal anomalies decreases rapidly and until the birth this rate reaches the level 0.5% or 1% (7).

chromosomal malformations can be divided into two categories: structural abnormalities and chromosomal number abnormalities, including loss or gain of chromosomes, called aneuploidy, or increased number of one or a complete set of haploid that is called polyploidy which could be due to errors in meiosis one or lack of segregation in meiosis two (2). Structural chromosomal abnormalities are the structural realignment of the chromosome that is the result of chromosomal failure and then composition in a different form which could be balanced or unbalanced. It is mainly caused by errors in meiosis. A discontinuous genetic variation that results in different forms or types of individuals among the members of a single species. The most common cytogenetic variants can be seen in satellites and short arms of acrocentric chromosomes that include the increase or decrease in the amount of heterochromatin of long arm of chromosome Y, inv, 16, 1, 9 (8, 2). These variations are generally called heteromorphisms because the term polymorphism is restricted by geneticists to a heritable variant that has a frequency of at least 1 percent in the population (2).

Most chromosomal abnormalities include number abnormalities such as monosomy, autosomal trisomy, triploid and tetraploid (6). Ward in his study showed that the risk of prenatal death of the monosomy embryo is 33% and for sex chromosome trisomy 13% and the death risk of trisomy 13 and 18 is 90% and Trisomy 21 is 12% (9). Estimation of different chromosomal abnormalities shows that if there is not any environmental difference, the prevalence in all pregnancies will be the same.

Any factor which can influence the frequency of survival and mortality has to been identified yet. While causing no abnormality in the appearance of people, inheritance abnormalities can highly influence population and cause to screening abortion and prenatal death mechanism (10). Balanced structural chromosomal abnormalities (including genetic material realignment, for example reversion and translocation, not complete loss or gain of genetic material) could be the cause of spontaneous abortion among the couples. 

In couples who have two or more abortions, the prevalence of these abnormalities is about 3-6% (5, 11). Couples with this abnormality can have a pregnancy with normal karyotype, or balanced or unbalance structural chromosomal abnormalities. This can lead to miscarriage, a stillborn child or a child born with severe congenital and mental defects (6). 

Common guideline for the management of spontaneous abortion is chromosomal analysis in both parents (8, 12). For counseling of the carrier couples about their risk for having children with chromosomal abnormalities or miscarriage or their chances for having a healthy child we need to have a statistical analysis results of generally population in similar anomalies. This counseling is mainly based on empirical estimates of risk and the history of reproductive or both of them in couple with reccurent abortion (1, 13). Although women with recurrent abortion who had 4 to 5 abortions may have a more dire prediction, because other factors may have participated in their abortion (11).

This study is a cross sectional descriptive study in which 75 couples with 3 or more abortions and 140 couples with 2 abortions before the 3rd month of pregnancy and also 40 control individuals have been investigated. Results of each group were compared separately with the results obtained from control subjects.

This study tries to investigate chromosome abnormalities and heteromorphism among couples with recurrent miscarriage in comparison to controls. The population that studied was individuals referred to gynecologist due to abortion, which were identified by the specialist and were referred to the cytogenetic laboratory for further evaluation.

## Materials and methods

This study is a cross sectional descriptive study. In this study patients were couples with recurrent abortions before 3rd months of pregnancy who were introduced to the cytogenetic laboratory of Shahid Beheshti Hospital in Hamedan since 2009-2011. The number of abortions and conditions were different. To study relationship between the number of abortions and frequency of chromosomal abnormality these couples were divided into two groups: those who had three abortions and more and couples with two abortions. Only the number of consecutive abortions among couples was studied. Their children were divided in to three groups: healthy children, abnormal children and no children. The study included a form containing questions which was filled by the couples after their consent to participate in this study.

Cultured peripheral blood lymphocytes of these people were used for chromosomal analysis and 5 ml of blood was cultured for these couples. In this study RPMI, HAM'S F10 culture medium subsequently was used to prepare culture area. We added 0.5 cc penicillin streptomycin solutions for each 50 cc, and 0.2 cc L-glutamine to boost the culture, and 0.2 cc nystatin for the antifungal effect.

This environment was sterilized by a filter. We measured PH of environment with ph meter and centrifuge 5 ml of heparin blood cell to separate red blood cells from white bloods. Then we removed red bloods. 6 drops of fetal calf serum (growth factor), 10 drops of patient's blood, and 3 drops of phytohemagelotin (stimulating the division) was added to each 5 ml vial of culture, then was put vials in water bath for 72 hours instantly. After 72 hours, we added to each vial 4 µg/ml Colcemid and put it in bath for 45 minutes then centrifuge it for 10 minutes, 1300 rpm. The supernatant was discarded and the sediment was kept. Then we added about 5 cc hypo KCL 7.5% to each vial or tube slowly and put the tubes for 20 minutes at 37^o^C water bath. Then we centrifuged tubes to 1,200 rpm for 10 minutes.

To each tube 5 ml of cold Fixative was added slowly and put them at room temperature for 20 minutes and then centrifuged to 1,200 rpm for 10 minutes and the supernatant was discarded. Again, we added about 5 cc of cold Fixative with Shaker to each tube. Then we centrifuged and discarded supernatant. Again we added Fixative and did the washing. This process was repeated until the solution became clear. After the planting and harvesting stages and slides, the glass slides were bonding by Gysma- trypsin. For each patient, there were fifteen banding metaphase microscopic study by analysor (asgari). In the cases with heteromorphism or anomalies more number of metaphase were examined. 

**Statistical analysis**

After collecting the information and determine chromosomal status of these individuals, the data was evaluated by SPSS software Ver. 18 and the frequency and type of heteromorphism and chromosomal abnormalities in couples in view of number of abortion was compared with controls.

## Results

Among 65 patients who were referred due to 2 consecutive abortions, 4 cases (6.15%) had chromosomal heteromorphism and 2 patients (3.07%) had chromosomal abnormalities. Also among 75 couples with three abortions and more, 7 cases (9.3%) had chromosomal heteromorphism and 4 cases (5.3%) had chromosomal abnormalities.

Different types of observed chromosomal abnormalities with the number of abortions and the number of their children and also heteromorphism types are shown in the table I and II.

In view of the number of abortions in couples ٬65 couples (46.6%) had two consecutive abortions, 12 couples (8.5%) had four consecutive abortions, three couples had five consecutive abortions (2.1%) and 60 couples (42.8%) had three consecutive abortions. 

Among couples with two abortions, 6 couples had abnormal children (9.2%) while 59 couples (90.7%) did not have abnormal children. Among these, 27 couples had healthy alive children (19.2%) and 38 couples did not have any healthy alive child. Among the couples with three abortions and more, 20 couples had healthy babies (26.6%), 7 couples had abnormal children (9.3%) and 48 couples (64%) had no children. In the survey of chromosomal abnormalities in women with two abortions, 1 woman (1.53%) had chromosomal abnormalities, 4 women (6.15%) had chromosomal heteromorphism and 60 women (90.23%) had a normal karyotype. 

According to the survey of chromosomal abnormalities among women with three abortions or more, 4 cases (5.3%) had chromosomal abnormalities and five of them (6.7%) had chromosomal heteromorphism. Also regarding to chromosomal abnormalities, among 65 men whom their wife had two abortions, 64 individuals had a normal karyotype and one had abnormal karyotype and two were heteromorphism. Also, among males whom their wife had three and more abortions one case (1.3%) had chromosomal abnormalities and two cases were with chromosomal heteromorphism (2.7%).

Comparison between the chromosomal status of men and women using Chi square test showed difference that this is significant in statistical analysis (p≥0.05), which represents higher chromosomal abnormalities among women than men.

Also in our study the frequency of chromosomal abnormalities in patients with recurrent abortion was compared to control group, that P in chi-square test of statistical analysis was equal to 0 (p≥0.05) and this indicates the possibility of chromosomal abnormality in miscarriage. Also, in a study to assess the presence or absence of the relationship between the number of abortions and frequency of abnormality in other children in this families it was determined that there is a significant relationship between these two parameters (p=0) (p≥0.05) and our study shows with increasing number of abortions increases the likelihood of chromosomal abnormalities.

Also in our study a comparison of the frequency of chromosomal heteromorphism were performed between control subjects (individuals without a history of abortion) and those with recurrent fetal abortions (p=0.114), (p≥0.05), that was not statistically significant .It shows that no relationship exists between abortion and chromosomal heteromorphism. Also in this study, 40 volunteers having no clinical symptoms were studied as control group to determine their karyotype. Only three cases (7.5%) of them were with chromosomal heteromorphism and 37 cases (92.5%) had a normal karyotype and no karyotype with chromosomal abnormalities were observed among these patients.

**Table I:** Types of abortions and number of chromosomal abnormalities observed in couples with repeated abortion

**Chromosome abnormality**	**Age**	**No. Spontaneous abortion**	**No. healthy children**	**No. abnormal offspring**
46, XX; t (13, 15) (q_10_ q_10_)	24	3	0	0
46, XY; t (7: 21) (q 11.2 q 22)	27	3	0	0
46, XX; t (8: 19) (q 24.1 q 13.1)	23	3	0	0
46, XX; t (13, 15) (q10 q10)	25	3	0	0
46, XX t (1: 12) (p22 q13)	25	2	0	0
46, XY; t (7: 21) (q 11.2 q 22)	35	2	0	0

**Table II:** Types of chromosomal heteromorphism and abortion and their children

**Chromosome heteromorphism**	**Age**	**No. Spontaneous abortion**	**No. healthy children**	**No. abnormal offspring**
46, XX; 16 qh+	23	3	0	0
46, XX; inv 9 (p11 q13)	34	3	1	1
46, XX; inv 9 (p11 q13)	24	3	0	1
46, XX; inv 9 (p11 q12)	27	3	0	0
46, XY; inv 9 (p11 q13)	31	3	0	0
46, XY; 15 ph+	25	3	0	0
46, XX; 22 stk+	28	3	0	0
46, XX; 16 qh+	23	2	0	0
46, XX; inv 9 (p11 q13)	17	2	0	0
46, XX; inv 9 (p11 q12)	20	2	0	0
46, XX; 16 qh+	37	2	0	0

**Figure 1:**
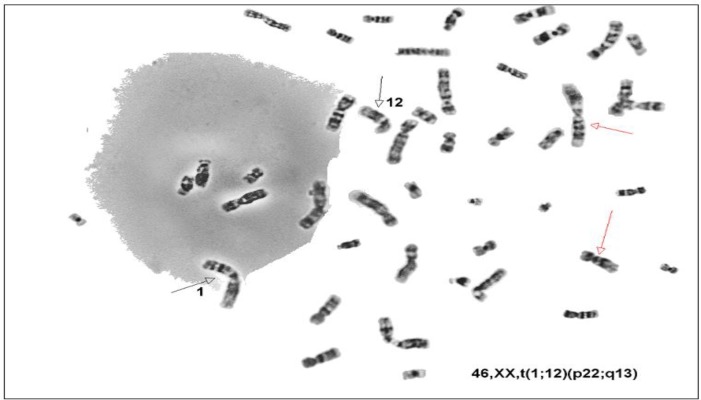
Translocation between chromosomes 12 and 1 in one woman with consecutive abortion.

**Figure 2:**
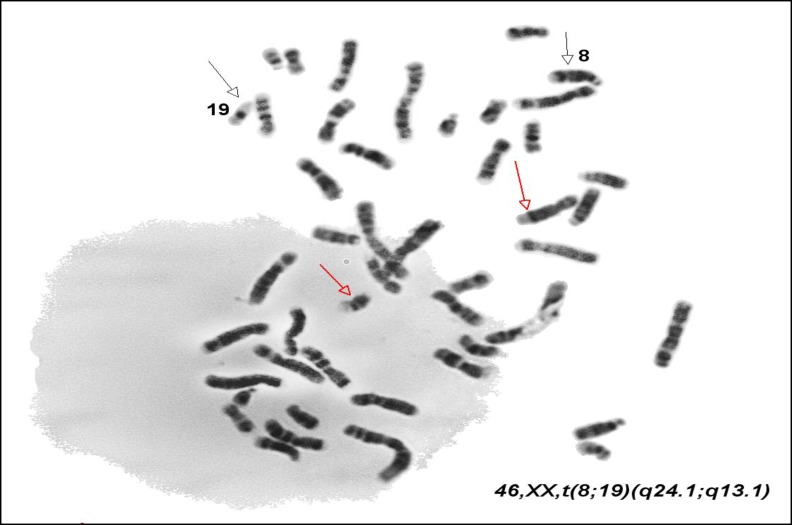
Translocation between chromosomes 8 and 19 in women with consecutive miscarriage.

**Figure 3:**
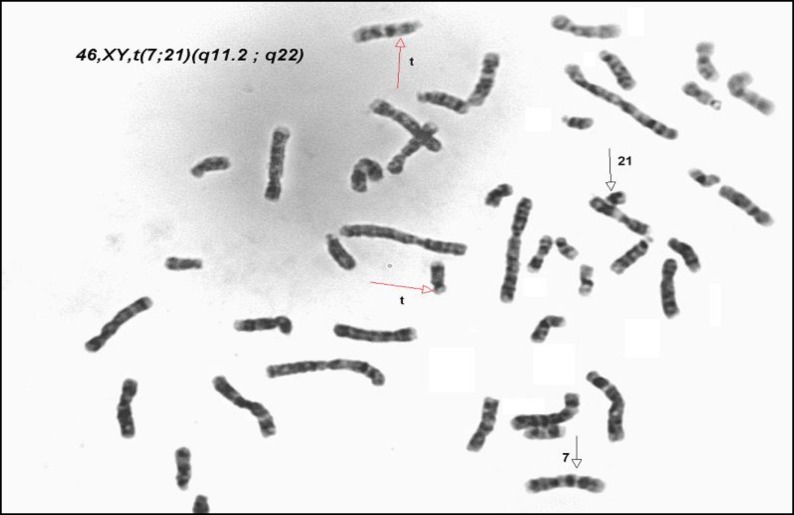
Translocation between chromosomes 7 and 21 in men whom his wife have recurrent abortion

## Discussion

Most of the spontaneous miscarriages are caused by chromosomal abnormalities in the embryo or fetus (14). The genetic factors represent more than 50% of early gestation spontaneous abortion and associated with fetal chromosomal abnormalities (15). The genetic etiology for multiple spontaneous pregnancy loss includes an unbalanced chromosome rearrangement, which may be the result of one parent being a carrier for a balanced chromosome rearrangement (14). 

In 4-8% of couples with recurrent pregnancy loss, at least one of the partners has chromosomal abnormality that probably contains balance chromosomal abnormalities (15). The prevalence of balanced translocation among couples with recurrent abortion in different studies ranges from 0-31% (3, 16). The reason of this extent variation is not clear. Prospective studies of couples identified as balanced translocation carriers, indicate that as many as 80% of their pregnancies end in spontaneous abortion, while only 16% lead to the birth of a healthy newborn. Their risk of giving birth to an abnormal child with chromosomal imbalance is relatively low approximately 4-6% (17).

Differences in the frequency of reports can be investigated due to differences in the investigation method as well. For example, in many of these studies control groups for comparison with the patients or the number of correct abortions, infertility or abnormal children are not considered. It makes difficult to compare these studies together. For example in the study of couples having more than two abortions no balanced translocation was found, while 4 of the 16 couples with two abortions associated with stillbirth or the children of a multiple congenital anomaly have balanced translocation. Apparently none of the couples with only two abortions were including in this study to be comparable with couples with malformed children who had two abortions (12).

In other study, translocations among 22 couples who were referred to genetic counseling with a history of recurrent abortion or birth of two or three stillborn children or malformed children was investigated, but the couples who had only abortions had not been considered (18). Michel also investigated among 200 couples with chromosomal translocation and reported frequency of balanced translocation was 6.3% (5). 

In Ferguson-smith study, couples were investigated in a genetics clinic and then referred to the cytogenetic laboratory, as we did in this study. Like this study they also excluded couples who had children with chromosomal abnormalities or stillborn children (13). There was a difference between Ferguson-smith study and our study. In Ferguson-smith study the incidence of abortion at any time during the reproductive history was investigated and consecutive abortions was ignored while in our study only the number of consecutive abortions of couples has been studied.

In Ferguson-smith study translocation carriers in two abortion groups was reported 8.4% and those who had three or more abortions found 5.4% but in our study the frequency of abortion in couples with two abortions was 3.7% and among the couples with three abortions and more found 5.3%. This was in the case that no chromosomal abnormalities were observed in control subjects. These results approximately agree to the findings of other researchers in different research centers (13). Thus it seems that racial difference has no significant effect in this regard. However, some differences can be seen in the percentages introduced by different researchers in different areas of the world (3, 13, 16).

Therefore it seems the prevalence of these abnormalities should be separately investigated in the different geographical areas (17). This shows that with increasing the number of abortions, the possibility of chromosomal abnormalities increases. Therefore, a cytogenetic study seems to be necessary for parents with recurrent abortions. Also because of considering the frequency of chromosomal heteromorphism in couples with recurrent abortion and controls, heteromorphism cannot be the reason for abortion in these patients. 

Also the possibility of chromosomal abnormalities in women is generally higher than men. Regarding to the close frequency of malformed children in both these couples, we can say that increasing the number of abortions in these couples will not increase the possibility of abnormal children but possibility of having healthy children will decrease in these couples. Detection of couples with chromosomal abnormalities can undoubtedly help to prevent the birth of malformed infants. 

Dewhurst *et al* also found a higher involvement of maternal X chromosome mosaicism, and reported that translocations of some chromosomes such as 1, 7 or 22 led to abortions, while those involving chromosome 5, 9, 14 or 21 led to the birth of handicapped children (3). However, it was seen that the involvement of chromosome 5, 9, 14 or 21 only led to miscarriages in our series. Their risk of giving birth to an abnormal child with chromosomal imbalance is relatively low approximately 4-6% (6). 

The exact risk depends on the specific chromosomes involved, size of the segment (s) involved in the rearrangement, sex of the transmitting parent, and mode of ascertainment (19). Nazmy (17) found a higher incidence of chromosome rearrangement in couples who had experienced both recurrent spontaneous abortion and viable pregnancies either normal or abnormal than in couples with recurrent spontaneous abortion and no viable pregnancies (20). Except chromosomal abnormalities, uterine deformities, endocrine disorders and infection can also cause abortion.

Eventually, our study agrees with several previous studies indicating an increase in the number of balanced chromosomal translocation in couples with two or more abortions compared with the general population. We concluded from all the previous results that cytogenetic studies should be performed to all couples with two or more spontaneous abortions and also in patients with recurrent IVF/ICSI failure. In a case of detected chromosomal aberration; the patient should be counseled individually according to the type of anomaly. This study should help physicians working in the region to realize the contribution of chromosomal abnormalities to cases of repeated fetal loss. It should also help in setting priorities of cytogenetic screening in individual cases (15).

The identification of chromosomal abnormality as the etiology has facilitated the counseling and appropriate management. Detailed cytogenetic analysis of both males and females with decreased reproductive fitness is essential for predicting the success of assisted reproductive procedures. Patients utilizing these emerging techniques need to be properly counseled as to their risks of transmitting these chromosomal abnormalities to their offspring. As the prognosis of couples having recurrent miscarriages may be good, even if one partner is carrying a translocation, the physician should encourage the couples, irrespective of their chromosomal status, to attempt for a healthy pregnancy (19).

Antenatal diagnosis can be offered to detect the foetal karyotypes, and pre-implantation genetic diagnoses with assisted reproductive technology are offered as management for repeated miscarriages in some centres (17). Even if such interventions are not available in some settings, the follow-up data of such couples with recurrent miscarriages would be useful to show the trend of possible future pregnancy outcomes. In the absence of data on the outcome of pregnancy, couples with balanced translocation can be informed only of the theoretical risk of abnormal pregnancies using hypothetical data (20, 21).
